# Ga-Doped ZnO Nanostructured Powder for Cool-Nanopigment in Environment Applications

**DOI:** 10.3390/ma13225152

**Published:** 2020-11-16

**Authors:** Ashraf H. Farha, Mervat M. Ibrahim, Shehab A. Mansour

**Affiliations:** 1 Department of Physics, College of Science, King Faisal University, Al Ahsa 31982, Saudi Arabia; 2 Department of Physics, Faculty of Science, Ain Shams University, Cairo 11566, Egypt; 3 National Center for Radiation Research and Technology, Atomic Energy Authority, Cairo 11566, Egypt; mervatibrahim@yahoo.com; 4 Basic Engineering Science Department, Faculty of Engineering, Menoufia University, Shebin El-Kom 32511, Egypt; shehab_mansour@yahoo.com; 5 Advanced Materials/Solar Energy and Environmental Sustainability (AMSEES) Laboratory, Faculty of Engineering, Menoufia University, Shebin El-Kom 32511, Egypt

**Keywords:** nanopigments, ZnO oxides, NIR reflectance, γ-irradiation polymerization, polymer-pyrolysis

## Abstract

Gallium (Ga) doped zinc oxide (ZnO) nanocrystals were successfully synthesized via a γ-radiation-assisted polymer-pyrolysis route. Ga doped ZnO samples with Ga and ZnO precursor salts with molar ratios of 0%, 3%, 5%, and 10% were produced. A γ-radiation dosage of 1.5 kGy was used for polymerization initiation during the sample preparation. The properties of the obtained nanocrystal samples were characterized using X-ray diffraction (XRD), Fourier transform infrared (FTIR), UV-visible absorption, NIR-VIS-UV diffused reflectance, and high-resolution transmission electron microscopy (HR-TEM) characterization techniques. XRD results revealed the formation of ZnO nanocrystals with wurtzite structure for both Ga-doped and undoped ZnO samples. Noticeable increasing in the line broadening of the XRD peaks as well as pronounced decreasing of crystallite size were observed with the increasing Ga ratio in the samples. Optical peaks around Ga:ZnO samples showed a blueshift in the optical absorption peaks with increasing Ga content. These results are in good agreement with the dependency of crystallites size as well as grain size on Ga ratio obtained from XRD and TEM images, which make them fit well for the powder cool-pigment applications. The doped samples showed high values of NIR reflectance ($R_{NIR}^{*}$) with percentage varied from 84.25% to 89.05% that enabled them to qualify for cool-nanopigment applications. Furthermore, such doped samples registered low values of visible reflectance ($R_{VIS}^{*}$) that enabled to reduce the glare from the reflected visible sunlight.

## 1. Introduction

Zinc oxide (ZnO) is among the II–VI semiconductors that have been extensively studied in recent years, because of its novel properties and broad applications. In general, ZnO-based compounds have gotten lots of attention due to their valuable properties, including their wide direct band gap (3.3 eV) at room temperature, high chemical stability, low processing cost, non-toxicity, and high-quality photoelectric and piezoelectric properties. Such numerous properties make ZnO extensively used in many applications [1,2]. The use of ZnO in industrial applications includes optoelectronic devices like liquid-crystal displays (LCDs), gas sensors, energy efficient windows of solar cells, and others [3,4]. Furthermore, ZnO as TiO_2_ has highly near-infrared (NIR) reflective feature for use in transparent conductive oxide applications [5,6,7]. Accordingly, significant interest of researchers is in the area of improvement of such ceramic materials for use as “cool” materials in painting applications [8,9]. In fact, more than the half of solar radiations are in NIR radiation region which is responsible for most of the heating effects of the solar energy. So, the use of materials with high transparency in the NIR region, such as ZnO as cool-pigments for painting the exterior surfaces of roofs, automobiles, etc., enables them to reduce the required energy for cooling such entities. Indeed, a significant absorption of the solar radiation via the dark surfaces of roads and buildings in large metropolitan areas causes an intensive increase in their temperature of about 10 °C in comparison with suburban and rural areas. This is due to the reemission of the formerly absorbed solar radiation at night. This phenomenon is called the urban heat island (UHI) effect [8,10,11]. The UHI phenomenon has massive consequences on the environment in two ways. One of them is the removal of the natural vegetation and the other is the intensive demand of energy consumption due to the wide usage of air conditioner (AC) systems in large buildings, especially in hot seasons. In addition to that, these AC systems are non-eco-friendly energy sources since the heat wastes produced by them also contribute to an increase in the UHI effect. Furthermore, this wide usage of the cooling systems could affect the ecological balance of the atmosphere. Consequently, this nanopigment painting using such eco-friendly materials has a larger impact on the limitation of UHI phenomena.

Pristine ZnO usually shows higher resistivity, lower transparency, and low carrier concentration affecting its performance in optoelectronic device applications [1,2]. Therefore, one of the ways to improve such poor electrical conductivity and low transparency of ZnO is to dope it using one or more of the Group III metal dopant elements, such as Indium (In), Aluminum (Al), and Gallium (Ga) [2,12,13]. Therefore, Ga:ZnO is considered one of the most important ZnO-based materials because it acts as a donor that could enhance the electrical properties of ZnO by increasing the free electron density, and/or carrier concentrations, [14,15]. Furthermore, the Ga ionic radius (0.62 Å) is smaller than that of Zn (0.74) and the shorter length of the covalent band length of Ga–O (1.92 Å) compare to that of Zn–O (1.97 Å) makes it simple for Ga^3+^ ion to substitute for Zn^2+^ ion with less lattice distortions and much increasing in the solubility of Ga ions into ZnO matrix [16,17,18,19]. 

Various preparation techniques have been employed for Ga:ZnO synthesization in powders as well as in film forms are such as sol–gel processing [19,20,21], pulsed laser deposition [22], magnetron sputtering [23], chemical spray pyrolysis [24,25], RF co-sputtering [26,27], polymer pyrolysis method [28] and many other methods. One of these methods and techniques which has lots of advantages compared to other synthetic chemical techniques is the polymer pyrolysis method. This method offers many of the advantages, such as lower processing temperatures, getting homogenously mixed precursors, lower cost, easy to control synthesization parameters, and requires a shorter calcination time compared to other methods [29]. 

Li et al. [28] reported the preparation of Ga doped ZnO nanoparticles by a polymer pyrolysis method. The polymerization of metal salts was promoted via an ammonium persulfate (APS) aqueous solution that was used as an initiator in the acrylic acid solution with heating at 90 °C. Modifications to a polymer pyrolysis method were added to obtain interesting properties for the obtained nanoparticles. In our previous work on Co:ZnO [30], two different polymerization initiators were used, namely ammonium persulfate (APS) and sonochemical (SON), during the synthesization of zinc oxide nanocrystals using a polymer pyrolysis method [30]. In this work, another modification is introduced, using γ-irradiation as an initiator to the polymerization process of the polymer pyrolysis route. To the best of our knowledge, this is the first report on the synthesization of Ga:ZnO nanocrystals using γ-irradiation as a polymerization initiator during the polymer-pyrolysis route. Consequently, this study aims to use the polymer-pyrolysis route via γ-radiation to synthesize Ga doped ZnO nanocrystals with Ga to ZnO salts molar ratio varied from 0 to 10 mol%. The effect of changing the Ga/Zn ratio in the samples on the structural, optical, and morphology features was investigated, as well as the suitability of the investigated Ga:ZnO nanopowders to be used in the cool nanopigment applications based on NIR solar reflectance (R*) calculations.

## 2. Experimental Details

### 2.1. Synthesization of Ga Doped ZnO Nanocrystals

**Figure 1:**
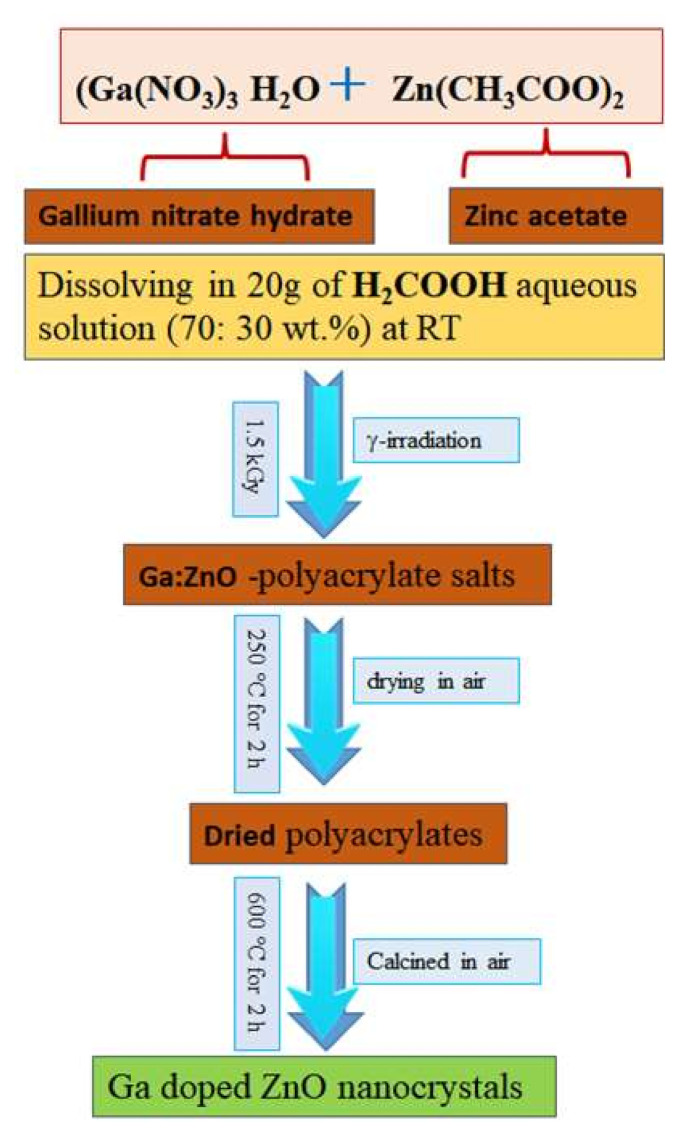
The schematic diagram of synthesis process of Ga doped ZnO nanocrystals using γ-radiation-assisted polymer-pyrolysis route.

In a typical route, gallium nitrate hydrate (Ga(NO_3_)_3_ H_2_O, (ABCR GmbH & Co. KG, Karlsruhe Germany) and zinc acetate dehydrate (Zn(CH_3_COO)_2_ 2H_2_O, Sigma–Aldrich, St. Louis, MO, USA) were dissolved in 20 g of acrylic acid (H_2_COOH, Merck, Millipore, Billerica, MO, USA) aqueous solution (acrylic acid: distilled water = 70:30 wt.%). The molar ratio of Ga(NO_3_)_3_:Zn(CH_3_COO)_2_ was varied from 0:100 to 10:100. The complete dissolving was done under stirring to form constantly distributed acrylate salts at room temperature (RT). Then, the solution was exposed to γ-radiation at 1.5 kGy followed by drying of the obtained polyacrylate at 250 °C for 2 h in air. According to the previous studies, the calcinations temperature for such compounds should be more than 500 °C [17]. Accordingly, all the dried polyacrylates were calcined at 600 °C for 2 h in air, which was less energy consumption process as a result of using γ-irradiation as an initiator for the polymerization process. The colours of the obtained by-product Ga doped ZnO powder samples are off-white with various degree of brightness. The samples were labelled as GZO-0, GZO-03, GZO-5, and GZO-10 that corresponding to the utilized molarity concentration ratio between Ga(NO_3_)_3_ H_2_O Zn(CH_3_COO)_2_ 2H_2_O; 0%, 3%, 5%, and 10%, respectively. Figure 1 shows detailed schematic diagram procedures for the synthesiszation process of Ga doped ZnO nanocrystals by using a γ-radiation-assisted polymer-pyrolysis route.

### 2.2. Characterizations 

The powder X-ray diffraction (XRD) measurements for the synthesized Ga:ZnO samples were done using X-ray diffractometer (MPD 3040, Philips X’Pert-MPD, Malvern, United Kingdom) with CuKα radiation source (λ = 0.15406 nm). The measurements were done throughout the diffraction angle (2θ) range from 10° to 80° with 0.02° step. The collected transmittance spectra of Fourier transform infrared (FTIR) for the investigated samples were obtained via a JASCO spectrometer (FT/IR-4100, Easton, MD, USA) through the wave numbers range between 400 and 4000 cm^−1^. The morphological characterization of the synthesized Ga:ZnO powdered samples was examined using high resolution transmission electron microscope (HRTEM, JEOL Ltd., Tokyo, Japan) Joel of type JEM-2100. The UV-VIS (ThermoFisher Scientific Inc., Waltham, MA, USA) and Photoluminescence (PL) measurements were achieved for synthesized Ga:ZnO powdered that was dispersed in ethyl alcohol at 1.5 mg/mL concentration. The UV-VIS absorbance measurements were measured throughout wavelengths between 200 and 800 nm using Thermo Scientific Evolution spectrophotometer of type 300 UV-VIS. Photoluminescence (PL) spectra were registered via Thermo Fisher Scientific-Lumina fluorescent spectrometer of type Waltham, MA, USA. The diffuse reflectance spectroscopic of the as-synthesized Ga:ZnO samples were tested using a UV/Vis/NIR spectrophotometer of type JASCO UV/Vis/NIR V570 with the incident wavelengths ranging from 200 to 2500 nm.

## 3. Results and Discussion

### 3.1. Structural and Morphological Characterizations of Ga:ZnO Nanocrystals

**Figure 2:**
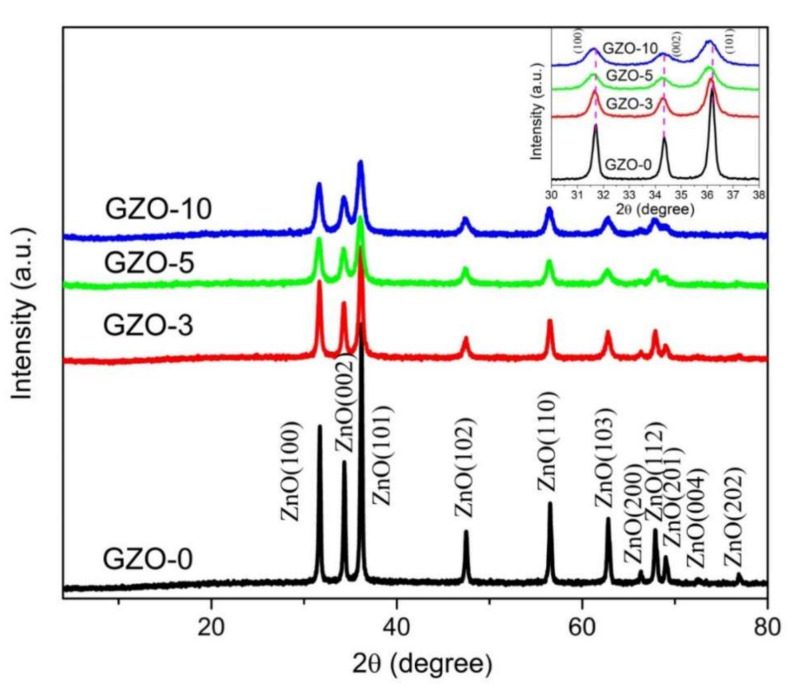
XRD patterns of as-synthesized Ga doped ZnO (GZO-0, GZO-3, GZO-5, and GZO-10) samples. Inset is an enlargement for the highest three peaks; (100), (0020) and (101); for more clarifications.

XRD measurements of Ga:ZnO nanopowder samples were done here to study the crystalline structure for as-prepared samples. Figure 2 shows XRD patterns of as-synthesized Ga-doped and undoped ZnO nanopowder samples with Ga:Zn various molar ratios. XRD patterns are exhibited the crystallization of Ga:ZnO nanocrystals in a wurtzite hexagonal structure for all the samples. The identification of peaks for the resultant XRD peak are shown on the patterns. All XRD peaks that identified in patterns for all investigated samples are matching with only a ZnO wurtzite structure peaks (JCPDS card 36-1451). The presence of a wurtzite ZnO structure with no other peaks of other ZnO phases or impurity phases of Ga or Ga oxides were identified in all samples. The characteristic diffraction peaks of as-synthesized Ga:ZnO samples that are corresponding only to wurtzite ZnO structure were also reported by many researchers worked on Ga:ZnO systems. Similar results were reported for Ga:ZnO nanoparticle samples prepared by a polymer pyrolysis method with Ga contents that are closer to those reported here c.f. [19,28].

Since (101) XRD peak showed the highest intensity compared to other XRD peaks in all the samples, the (101) direction along the c-axis is the preferential growth direction for all as-synthesized Ga:ZnO samples. Alternatively, the intensities of diffraction peaks of the Ga-doped samples reduced as Ga contents in the sample was increasing as it is clearly seen in inset of Figure 2. Here the inset shows magnification for the highest-intensities three XRD peaks with orientations (100), (002) and (101) of all the samples. The decrease in the intensity of XRD peaks with increasing metal element concentration (Ga) in the samples is due to slowing down of ZnO growth as Ga concentration increases in the samples [31].

Increasing the Ga content in the samples clearly affects the crystallinity of the nanocrystal samples as well the position of XRD peaks of Ga doped ZnO compare to pure ZnO sample. A visible shift in the position of XRD peaks to low 2θ values with increasing Ga concentration in the samples can be seen in the inset of Figure 2. Such shifts to lower 2θ values indicate an increment in the volume of the unit cell as Ga content in the samples is increasing [32]. This conclusion is confirmed by increases in the calculating volumes of the unit cell that were obtained for Ga:ZnO nanocrystal samples and summarized in Table 1. This increase in the cell volume with Ga increases can be explained because of the differences in the ionic radii of Ga and Zn ions which causes such increase in the cell volume [33]. Table 1 is listing the structural parameters that were calculated from the XRD results for Ga:ZnO nanocrystals samples in accord to ZnO wurtzite unit cell. The parameters listed in Table 1 include: the crystallites size (D) calculated using known Scherer formula, the lattice constant wurtzite cell (c & a), (c/a) ratio, the volume of the cell (V), and the strain (ε) of Ga doped ZnO samples. No pronounced changes in values lattice constant a were observed while lattice constant c was showing very little increasing than value of pure ZnO sample. These results are supporting the proposal for the substitution of Ga to the Zn sites in the ZnO matrix [17,33]. 

Moreover, Table 1 showed the average strain values calculated from strain equation [34],

(1) $$\varepsilon =\;\frac{c-c_{o}}{c_{o}}\;\times 100\%,$$

where *c* is lattice constant for each sample as calculated from XRD results and *c_o_*_,_ known as strain-free lattice parameter and it equals to 5.206 Å for the bulk ZnO. All samples showed tensile strain, as indicted by the obtained positive strain values as given in Table 1. The values of the strain is increasing as Ga content in the samples is increasing. The average crystallite size values, *D*, were calculated using Scherrer method:

(2) $$D=\frac{0.94\;\lambda }{B_{hkl}\cos\theta },\;\;$$

where λ, *B_hkl,_* and θ are the wavelength of used X-ray beam (1.5406 Å), the full width at half maximum (FWHM) of the XRD peak and θ is the Bragg diffraction angle at (*hkl*) plane, respectively. The average crystallite size in Table 1 is showing a deceasing trend as the Ga content is increasing in the samples. Here, it is worth mentioning that the reduction of the crystallite size is due to the high crystalline solubility as Ga content in the samples increases. High strain values that obtained with increasing dopants in the samples induce higher surface energy for the nanocrystals and that Ga dopants hinder the growth of ZnO crystals [35].

**Figure 3:**
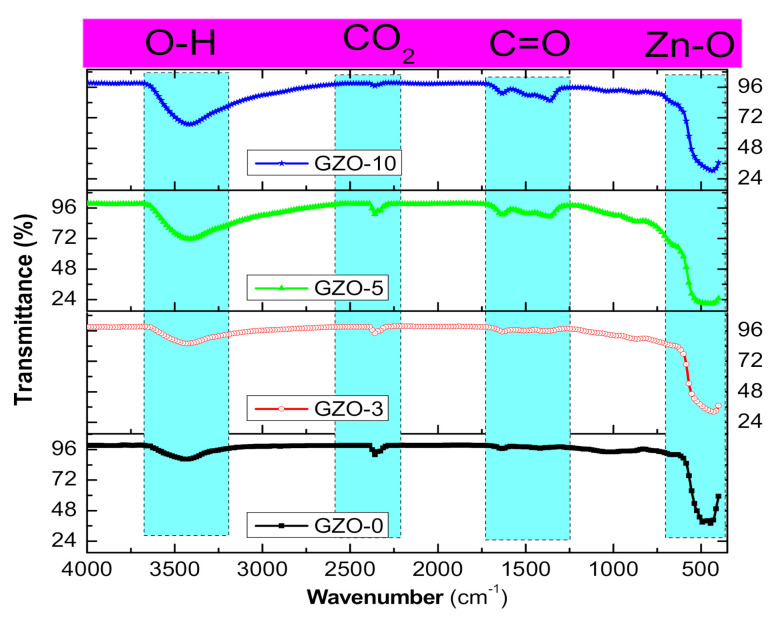
The absorption FTIR spectra as-synthesized Ga doped ZnO (GZO-0, GZO-3, GZO-5, and GZO-10) samples.

For more exploration on the structural pattern of Ga:ZnO samples FTIR analysis were done. FTIR transmittances for the samples are depicted in Figure 3 with identifications of observed peaks. The obtained FTIR results are supported the suggestion that made from the XRD results for the formation of wurtzite structures in all samples. The FTIR bands that emerged in all spectra of the samples are as follows: the O–H vibration bands visible at about 3430 cm^−1^. The intensity of such O–H absorption bands that are associated to the O–H mode is increasing as Ga content in the samples is increasing. This may be attributed to the decrease in the particle size that leads to a probable increase in OH adsorption due to the increase in surface area. This result is in good agreement with the crystallite size obtained from XRD measurements. In further investigations for the OH contents in the samples, the ratio between the intensities of O–H and Zn–O absorption peaks were found and are listed in Table 1. The OH/ZnO ratio is clearly showing some increases as Ga content increases in the samples. Such increases are a result of the decrease in the surface area of the nanocrystals. Hence, the less the size of particles, the larger its surface area, which causes great numbers of OH groups to desorbed on the surface. The absorption band at 2360 cm^−1^ which is ascribed to CO_2_ may be because samples are getting it from the atmosphere. The C=O two asymmetric and symmetric stretching bands at 1430 and 1640 cm^−1^ are resulting from the zinc acetate in the samples. The absorption band that appears at about 440 cm^−1^ can be assigned to the ZnO stretching modes [36]. The ZnO stretching mode is showing small changes which is reflecting on the unpronounced changes seen in the volumes of ZnO cell. This is in good agreement with cell volumes that were calculated from XRD results. 

**Figure 4:**
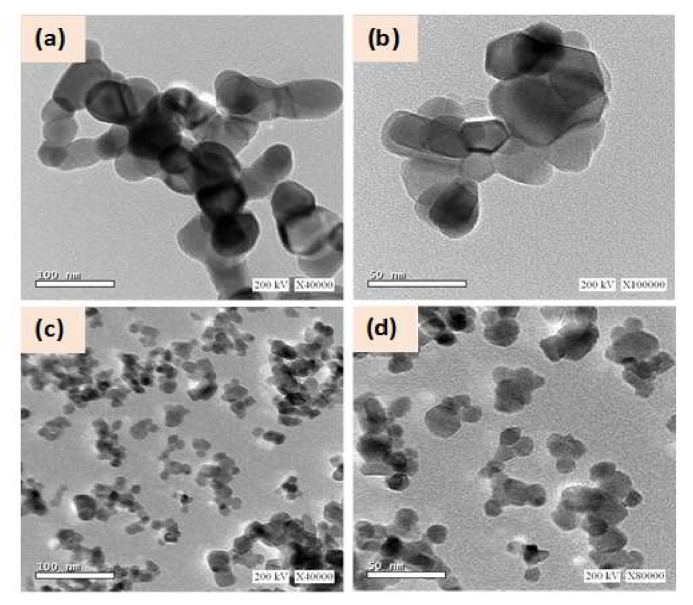
HRTEM micrographs for the investigated Ga-doped ZnO; (**a**,**b**) for GZO-0 and (**c**,**d**) for GZO-5.

Figure 4 shows the HRTEM micrographs for the GZO-0 and GZO-5 samples. Such micrographs clarify the morphology and particle size between undoped ZnO sample and doped sample. Specifically, both of such samples have particles with spherical-like shape in nanostructure. Whereas, the agglomeration of particles is higher in case of GZO-0 sample than in case of GZO-5. Furthermore, the particle size varied from 30 to 70 nm and from 10 to 20 nm for GZO-0 and GZO-5, respectively. The lower particle sizes of the doped sample (GZO-5) than that obtained for pure sample (GZO-0) are in agreement with the results of the crystallite size as obtained from XRD data. Here it is worth to mention, the effect of using γ- irradiations as polymerization initiator in polymer-pyrolysis method by comparing the results that obtained here with our previous work on ZnO nanoparticles [30]. In that work ZnO nanoparticles were synthesized by polymer-pyrolysis route using two different polymerization initiators, namely ammonium persulfate (APS) and sonochemical (SON) as polymerization initiators. APS gives mixed types of particles (hexagons and rods) shaped-particles with higher aggregation, while here particles are one-type of a round shape. Regarding the particles size, one noticed the size of current ZnO sample (30–70 nm) lies between smallest size for ZnO nanoparticles that was obtained by APS (15–45 nm) and that obtained by SON (58–120 nm) [30] with less aggregations than both cases as well as that reported for ZnO synthesized by polymer-pyrolysis [28]. Certainly, the particles size had a tendency to increase and their aggregations are changing upon irradiations as reported for the effect of γ-irradiations [37] on the morphology of ZnO nanopowders.

### 3.2. Optical Characterization

**Figure 5:**
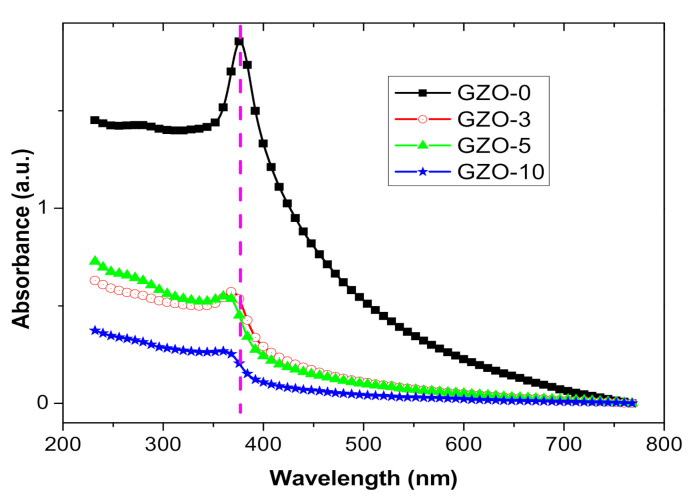
UV-Vis spectra of as-synthesized Ga doped ZnO (GZO-0, GZO-3, GZO-5, and GZO-10) samples with different gallium nitrate additions.

Optical absorption spectra of Ga:ZnO samples that dispersed in ethanol solution are shown Figure 5. All samples are illustrating a strong absorption peak, with no any additional peaks in the spectra which affirms no formations of impurities are existed in the samples [30,35]. The absorption spectra of the Ga:ZnO samples demonstrated blueshifts at the absorption band edge as Ga content increases in the samples. The peak position values of 378, 368, 362, and 360 nm were indicted for GZO-0, GZO-03, GZO-5, and GZO-10 samples, respectively. Such a blueshift of the absorption edge to lower wavelengths is indicating increase in the carrier concentrations due to the Ga^3+^ substitution for Zn sites [35]. These created charge carriers tend to occupy the lowest states of the conduction band and as a result widening of the band gap is expected to occur this is so-called Burstein–Moss (BM) effect [35,38]. The increasing of the band gap with the Ga content occurs till some specific concentration after that it starts to decrease. The narrowing of the band gap results from Coulomb interactions between the free carriers as well as from carrier-impurity scattering which are causing shifts in both the conduction band the valence bands toward lowering the band gap [35]. Here, it is worth mentioning that the nanoparticles` size has an insignificant effect on the obtained band gap values since the obtained nanoparticle sizes are much bigger than the radius of Bohr extions (2.1 nm) [35]. The present results are in good agreement with the achieved changes in the particles size with Ga concentration that were obtained from TEM measurements. The band gap of the nanoparticles increases with the decreasing of the particle size. Similar results were reported for Ga doped ZnO nanocrystals [35,39]. 

**Figure 6:**
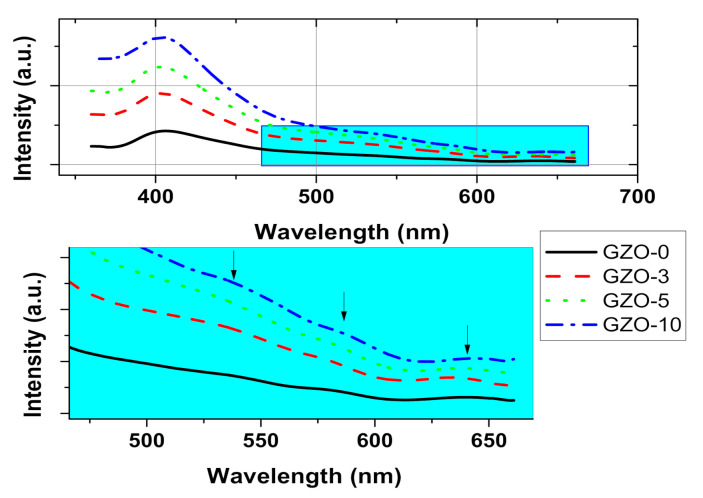
PL spectra of as-synthesized Ga doped ZnO (GZO-0, GZO-3, GZO-5, and GZO-10) samples @ excitation wavelength of 350 nm.

Photoluminescence (PL) spectroscopy were preformed deeper investigations on the effect of the defect states upon the optical properties as the Ga doping concentration changes in the samples. Figure 6 shows PL spectra of Ga doped ZnO nanocrystals that obtained upon an excitation with 350 nm wavelength UV light. All samples showed both near band and defect level emissions. Band to band emission is not seen in PL spectra for all samples the absence of this emission is related to the excess oxygen vacancies on the surface of ZnO nanoparticle [40]. The near band emissions (NBE) are in the UV range around 402 nm which are corresponding to the near recombination of shallow-trapped charge carriers near the conduction and valence bands. The difference between obtained values for NBE investigated samples (~402 nm) and the expected value (~370–380 nm) can be attributed to the electrons transition in the localized tail levels [30,41]. The intensity of these emissions is falling off and they also showed a blueshift as Ga content in the sample is increasing. The other emissions in visible region, defect level emissions, are resulting from charge carriers recombination in the defect levels within the band gap [42]. These emissions appeared over a wide visible region ranging from 530 to 640 nm, namely green, yellow, and orange-red emissions with clear weaker intensities than near band emissions as shown in Figure 6. The visible region emissions could be resulted from large varieties of defects since the defect formation as well as defect concentration in Ga doped ZnO are very complex [35,42]. The relative intensities of near band peaks to that of the defect levels are decreasing with Ga concentration in the samples, which means the electron–hole recombination occurs in the deep defect states rather than near band states [35]. Such a result confirms a rising in the number of carriers in the defect states with Ga addition. The relative intensity of the PL emissions is showing tiny changing upon Ga additions since our doping in the samples is very close to the solid-solubility limit in ZnO [43]. Recalling the effect of using γ-irritations, here, the PL spectra of GZO-0 experience an absence of the blue emission in comparison to APS or SON polymerization initiators on PL emissions of our previous work on ZnO nanoparticles [30]. Here, it is worth mentioning that the absence of blue emission could be attributed to the O-deficient defects. In contrast, the blue emission is related to donor defects, i.e., oxygen vacancy (V_O_) and/or interstitial zinc (Zn_i_) [44]. Such a result is in a good agreement with the common effect of γ radiation in the promotion of oxygen deficiency [45].

## 4. UV-Vis-NIR Diffuse Reflectance Spectroscopic Characterization of the Synthesized Ga-Doped ZnO Nanocrystals

**Figure 7:**
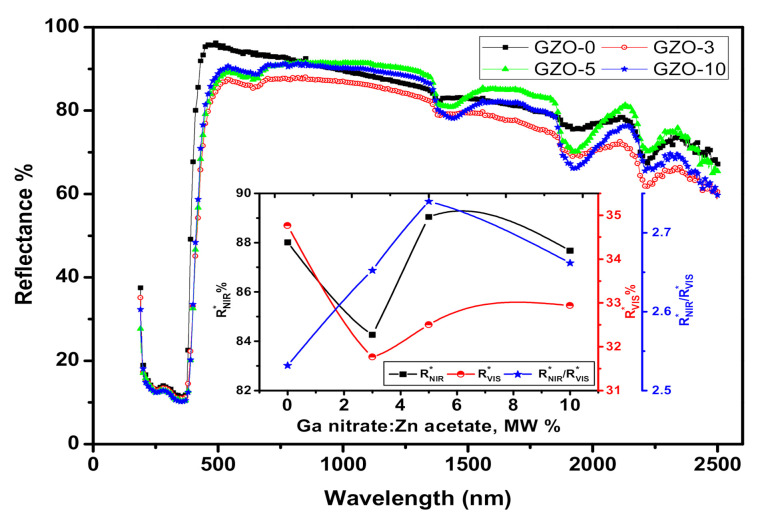
UV/vis/NIR diffuse reflectance spectra of the investigated Ga-doped ZnO nanopigments. The inset represents the variation of $R_{NIR}^{*}$, $R_{VIS}^{*}$ and $\frac{R_{NIR}^{*}}{R_{VIS}^{*}}$ as a function of Ga nitrate molarity weight percentage.

**Figure 8:**
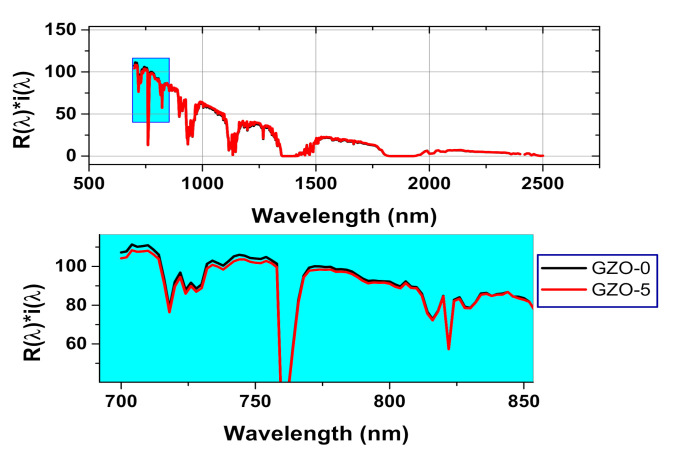
The effective reflectance, $R\left(\lambda \right)$ *$i\left(\lambda \right)$ spectrum for GZO-0 and GZO-0 nanopigment samples using a real incident solar radiation according to the ASTM (G173-03).

Figure 7 shows the DRS spectra of the investigated Ga-doped ZnO nanopowders in range of UV-Vis-NIR. All the investigated nanopowder samples showed strong band absorption at wavelength values less than 370 nm that are related to the originated optical transitions from optical energy gap [46]. The obtained maximum value of reflectance (*R*) was reached to ~96% at 484 nm as recorded for the pure ZnO, GZO-0, and then *R* values showed approximately linear-inversely proportionality with λ up to 1350 nm with *R* value at about 85%. In contrast, the maximum values of R that were recorded for the doped samples were around 90.5, 89.5, and 87.5 at 540 nm for GZO-3, GZO-5, and GZO-10, respectively. The DRS spectra of doped samples were exhibited small drops of R values in λ range from 541 to 688 nm which could be arising from absorption peak related to the probable existence of other phases based on Ga. On contradiction to the pristine sample, the doped samples exhibited almost reflectance plateaus in λ range from 688 to 1300 nm with R values ~91.5%, 90%, and 87% for GZO-3, GZO-5, and GZO-10, respectively. In order to examine the suitability of the investigated nanopowders in cool nanopigment applications, NIR solar reflectance (R*) was calculated. As is known, R* is considered one of the figures of merit for determining the cool performance of the pigments. Specifically, NIR radiation ranging from 700 to 2500 nm is representing ~52% of the solar energy radiation [47], which is considered one of the basic reasons for buildings heating up. In this respect, R* values of the synthesized Ga-doped ZnO nanopowder samples were obtained throughout λ range from 700 to 2500 nm according to the ASTM (G173-03) and the following formula [48]:

(3) $$R_{NIR}^{*}=\frac{\int_{700}^{2500}R\left(\lambda \right)i\left(\lambda \right)\;d\lambda }{\int_{700}^{2500}i\left(\lambda \right)d\lambda }$$

where $R\left(\lambda \right)$ is the measured spectral reflectance (Wm^−2^) and $i\left(\lambda \right)$ is the solar spectral irradiance (Wm^−2^ nm^−1^) as registered under ASTM standard G173-03 conditions. The variation of $R_{NIR}^{*}$ as a function of Ga content is illustrated in the inset of the Figure 7. The obtained values of $R_{NIR}^{*}$ varied from 84.25% to 89.05% and they are considered high values. This is suggested the ability of the used preparation technique to obtain ZnO-based nanopowders with highly NIR reflectance for cool nanopigment applications. The obtained $R_{NIR}^{*}$ value for the pure ZnO samples, ~88%, is considered quite high value in comparison to that obtained for ZnO prepared using other techniques such as arc discharge and thermal decomposition techniques [49,50]. Furthermore, GZO-5 sample exhibited highest $R_{NIR}^{*}$ value ~89.05% even higher than the pure ZnO sample, GZO-0. This result could be attributed to the small particle size of GZO-5, 13.7 nm, that offers a compensate effect against the increase in the trap states due to doping by Ga [51]. Figure 8 shows the effective reflectance spectrum, $R\left(\lambda \right)$*$i\left(\lambda \right)$, throughout the NIR range from 700 to 2500 nm for both undoped ZnO sample (GZO-0) and the doped sample (GZO-5) with 5 MW% of Ga nitrate. The spectra of these samples are showing very close values without a specific trend in the whole range of wavelength. Specifically, $R\left(\lambda \right)$*$i\left(\lambda \right)$ spectra of GZO-0 sample has higher values than that of GZO-5 in the low range of wavelength from 700 to 850 nm as shown in the zoomed part of spectra in Figure 8. Whereas, this trend has been reversed in the high range of wavelength from 850 to 2500 nm as seen in Figure 8. Such non-monotonic trend refers to the competition between the increase in the effective reflectance due to high scattering feature of small particle size and the expected limited trap sites in case of GZO-5 and GZO-0, respectively. The slightly higher value of the cooling performance parameter ($R_{NIR}^{*}$) for GZO-5 than that which corresponded for GZO-0 is required to be checked through another parameter that is related to aesthetic performance, namely ($R_{VIS}^{*}$). $R_{VIS}^{*}$ is measuring the effective reflectance in visible range of light with taking in account the spectral sensitivity of eye for the reflected light in the visible range. Accordingly, $R_{VIS}^{*}$ values of the investigated samples were estimated throughout the wavelength range from 390 to 700 nm using the following formula [52]:

(4) $$R_{VIS}^{*}=\frac{\int_{390}^{700}R\left(\lambda \right)\eta \left(\lambda \right)i\left(\lambda \right)\;d\lambda }{\int_{390}^{700}i\left(\lambda \right)d\lambda }$$

where $\eta \left(\lambda \right)$ is the normalized standard luminous efficiency under photopic situation of CIE (international commission on illumination) #1931 [53]. The variation of the estimated $R_{VIS}^{*}$ values for the investigated Ga-doped ZnO samples is shown in the inset of Figure 8. This figure shows that GZO-3 has the lowest value of $R_{VIS}^{*}$ with respect to the other studied samples. Furthermore, all the doped samples have lower $R_{VIS}^{*}$ value than that estimated for the pure ZnO sample. This decrease in $R_{VIS}^{*}$ value is referring to the increasing in the absorbance in visible range for the doped samples due to the trap sites that correlated to the Ga addition. Here, it is worth mentioning that, the low values of $R_{VIS}^{*}$ offer a reduction of the glare of the reflected sunlight from such materials in the case of utilizing them in nanopigment coating applications. The reduction in the glaring of the reflected sunlight from the coating nanopigment materials enables to reduce the visual discomfort. In addition to that, an expected reduction in the usage of the electrical power in cooling systems is consequential and will has am optimistic effect on the ecological balance in the atmosphere. In fact, the design of the cool nanopigments in coating applications entails optimization to enhance the aspects of the cooling feature and the aesthetic feature to obtain high value of $R_{NIR}^{*}$ and low value of $R_{VIS}^{*}$, respectively [52]. So, the ratio between $R_{NIR}^{*}$ and $R_{VIS}^{*}$ should be maximized. By calculating $\frac{R_{NIR}^{*}}{R_{VIS}^{*}}$ for the studied samples, GZO-5 sample showed the highest value of this ratio as seen in the inset of Figure 8. This result suggests that Ga content in GZO-5 sample using the used preparation technique offered optimum reflectance behavior to be used as white cool nanopigment for both of cooling and aesthetic sides.

**Figure 9:**
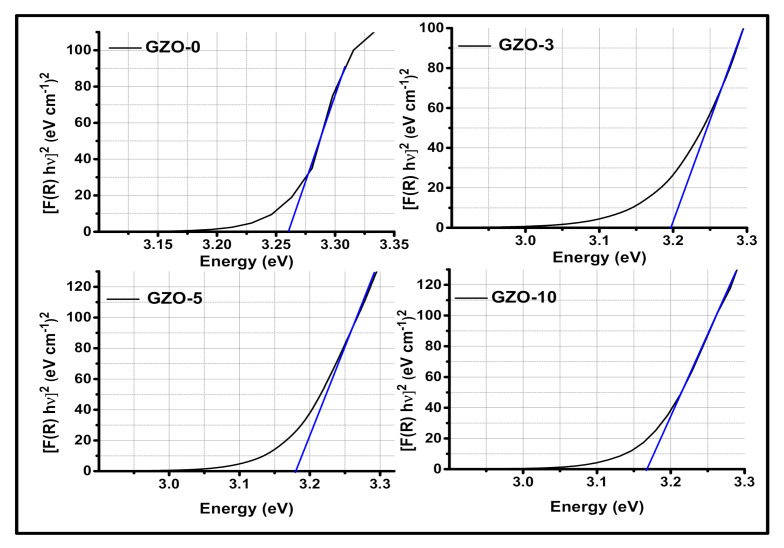
Plots of ${\left[F\left(R\right)h\nu \right]}^{2}$ versus Energy = hν for the investigated Ga-doped ZnO nanopigments.

**Figure 10:**
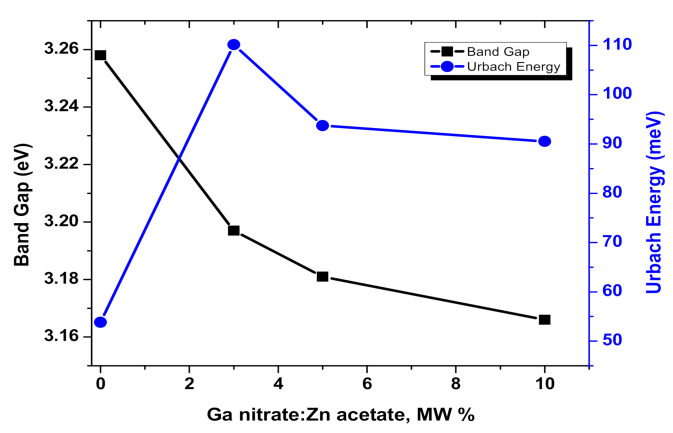
Band gap and Urbach energy variations as a function of Ga nitrate molarity weight percentage for the investigated Ga-doped ZnO samples.

The band gap (*E_g_*) is commonly obtained for powdered materials from DRS results using the Kubelka–Munk (K-M) approach. Accordingly, K-M function *F*(*R*) could be evaluated by using the following formula [54]:

(5) $$F\left(R\right)=\frac{{\left(1-R\right)}^{2}}{2R}=\frac{k}{s}$$

where *R* is the recorded diffused reflectance from the powder samples. However, k and *s* are called the K-M absorption and scattering coefficients, respectively. Indeed, the K-M was suggested that *F*(*R*) is equivalent to the linear absorption coefficient (α). The general relation α and *E_g_* for direct band gap semiconductors is given by the following formula [46,55]:

(6) $${\left(\alpha h\nu \right)}^{2}=C_{1}\left(h\nu -E_{g}\right)$$

where hν is the incident photon energy on the studied sample and $C_{1}$ is the a proportionality constant. Under the optimum diffuse condition and the incident angle of illumination at 60°, *k* can be considered equal to the twice of *α* value. Furthermore, the independency of *s* factor on the wavelength of the incident light suggested that the Equation (6) could be rewritten using *F*(*R*) as the following [46,55]:

(7) $${\left[F\left(R\right)h\nu \right]}^{2}=C_{2}\left(h\nu -E_{g}\right)$$

where $C_{2}$ is constant related to s and $C_{1}$ factors. Accordingly, Figure 9 shows plots of ${\left[F\left(R\right)h\nu \right]}^{2}$ versus hν for the investigated nanopowder samples. *E_g_* value was determined for each studied sample at the intersection between the extrapolation of the linear portion of the curve at ${\left[F\left(R\right)h\nu \right]}^{2}=0$. The obtained *E_g_* values were decreased with increasing Ga content as shown in Figure 10. This reduction of effective band gap for the doped samples could be attributed to both of the sub-band states of Ga^3+^ and oxygen defects [56]. Furthermore, *E_g_* for all the investigated samples, varied from 3.166 to 3.258 eV, is a small value in comparison with the case of bulk ZnO (3.37 eV). This can be attributed to the high density of the chemical defects and/or vacancies at the intergranular regions that is caused a decrease in the band gap [50,57,58].

**Figure 11:**
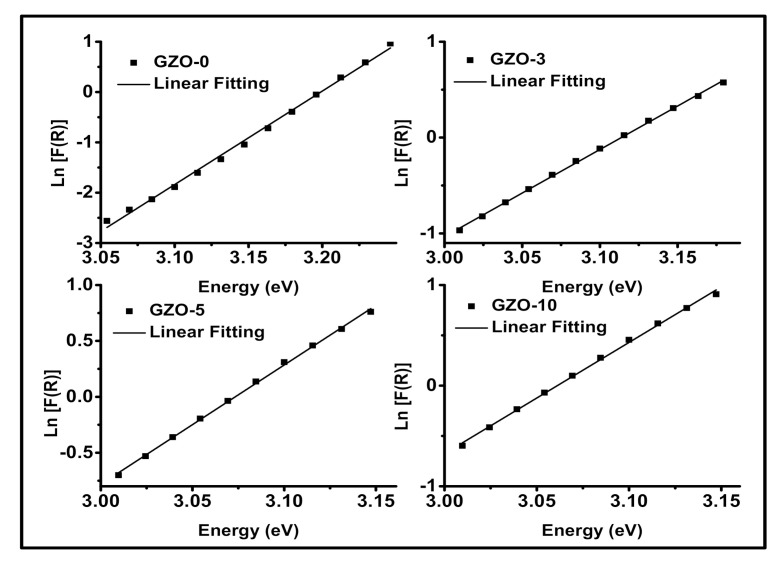
Plot of ln [*F*(*R*)] versus Energy = hν for the investigated Ga-doped ZnO nanopigments.

The existence of defects and/or vacancies due to doping by Ga leads to the formation of band gap states which emerged as band tail extending on both edges of valence band and conduction band. Such extended states of defects are known as the Urbach tails and the energy of this tail is named as Urbach energy (*E_U_*) [56]. As known, the E_U_ could be evaluated using the following formula of the absorption coefficient [59]:

(8) $$\alpha =\alpha_{0}\;\exp\left(h\nu /E_{U}\right)$$

where $\alpha_{0}$ is the pre-exponential factor. According to the K-M assumptions, *F*(*R*) is equivalent to *α*. So, in the current study the E_U_ could be determined by plotting $\left[\ln F\left(R\right)\right]$ versus the incident photon energy (hν). Here, the plot of $\left[\ln F\left(R\right)\right]$ versus photon energy gives quite good straight line for each studied samples as shown in Figure 11 and the *E_U_* values are the reciprocal of the slopes. The variation of E_U_ values as a function of the Ga content is shown in Figure 10. The *E_U_* values are varied from 53.8 to 110.14 meV and the highest value of E_U_ is obtained for GZO-3 sample. This result supports the lowest value of $R_{VIS}^{*}$ as previously discussed and shown in the inset of Figure 7. Here it worth to mention, the decreasing in $R_{VIS}^{*}$ could be attributed to the high density of trap states that leads to increase in the absorbance in the visible range.

## 5. Conclusions

Undoped and Ga-doped ZnO nanocrystalline powders have been synthesized by a γ-irradiation assisted-polymer pyrolysis route. The microstructure characterization confirmed the invariability in the wurtzite structure of ZnO for all the synthesized doped samples. The addition of Ga has a significant effect on the limitation of ZnO growth which is reflected in the pronounced decrease in the size of particles with the increase in Ga concentration. The blue emission is almost absent for the synthesized samples which confirms the role of γ-radiation in increasing the O-deficient defects that responsible for such emission in of Ga:ZnO. The defects due to the doping by Ga are also responsible for the narrowing of the band gap of ZnO by formation of Urbach tail on both edges of valence band and conduction band. The obtained small particle size of the doped samples as well as their low values of visible reflectance ($R_{VIS}^{*}$) enable such samples to be qualified as cool nanopigments in view of their cooling and aesthetic performances especially the GZO-5 sample. Hence, the currently investigated Ga doped ZnO functional materials can be preserved among the eco-friendly cool-nanopigments due to their expected role in the reservation of environmental balance by limiting the UHI passive phenomenon.

## Figures and Tables

**Table 1:** Crystallites size (D), the wurtzite ZnO lattice parameters (c & a), cell volume (V), average values of the strain (ε) along the c-axis and (c/a) ratio for Ga doped ZnO samples and the ratio between the intensity of the FTIR absorption bands of OH to ZnO.

Sample	D, nm	a, Å	c, Å	V, Å	c/a	ε
GZO-0	31.5	3.254	5.214	47.820	1.602	0.156
GZO-3	17.4	3.257	5.212	47.896	1.600	0.132
GZO-5	13.7	3.267	5.226	48.302	1.599	0.389
CZO-10	10.9	3.261	5.240	48.248	1.603	0.654
